# Students' perceptions of a flipped classroom approach to paramedic theory

**DOI:** 10.29045/14784726.2018.03.2.4.1

**Published:** 2018-03-01

**Authors:** Sarah V. E. Christopher

**Affiliations:** University of Lincoln

**Keywords:** allied health personnel, learning, perception

## Abstract

**Introduction:**

The flipped classroom is a pedagogical model in which the typical lecture and homework/self-study elements of a course are reversed or ‗flipped‘. The flipped model differs from distance and online learning, since students still have face-to-face contact with tutors. Studies have shown that the flipped classroom approach can be effective with clinical skills learning by paramedics, but it is not clear whether anatomy, physiology, pathophysiology and pharmacology can effectively be taught using a flipped approach.

**Methods:**

A flipped classroom approach was taken for a three-hour theory session on the Diploma of Higher Education in Paramedic Practice that would usually have been taught by traditional lecture. Participants, 20 student paramedics already employed by the Ambulance Service as Emergency Care Assistants, completed a two-part questionnaire after the session. Part one comprised 22 Likert style questions, with part two allowing qualitative data with five open ended questions.

**Results:**

All students preferred the traditional lecture over a flipped approach. Time constraints and preparation were seen as major disadvantages of the flipped classroom, as was being unable to ask questions in real time. Flexibility was seen to be an advantage of online lectures, and a blended/combination approach of online resources and traditional lecture was seen as valuable.

**Conclusion:**

The demographic of students may have had an impact upon the results of the study and the lack of popularity of the flipped approach. Employed students have the added pressure of full-time work, and further research is needed into whether different methods should be employed when choosing how to educate employed paramedic students versus those who enter via the undergraduate route and study full time.

THE FLIPPED CLASSROOM THE STUDENT PERCEPTION QUESTIONNARIE Please use the extra paper provided for the free text questions if necessary

**Table:** 

	Question	Strongly disagree	Disagree	Neither agree nor disagree	Agree	Strongly agree
1	The flipped classroom model is more engaging than traditional classroom instruction					
2	I like watching lectures on video					
3	I find it easier to understand subjects after attending a traditional lecture					
4	I would not recommend the flipped classroom to a friend					
5	I would rather watch/listen to a traditional teacher-led lesson than a video					
6	The flipped classroom enabled me to understand the subject better					
7	I find class activities of more value to my learning than a lecture					
8	I feel I benefit from discussions that arise spontaneously in lectures					
9	It is the responsibility of the lecturer to ensure I learn					
10	I am in charge of my own education					
11	I like being able to work at my own pace					
12	I like flexibility in my learning					
13	I prefer a structured approach to my learning					
14	I like the tutor to set the pace of my learning					
15	The flipped classroom model means I am spending more time on self-study					
16	I find it easier to learn in my own time rather than in a lecture					
17	I spend more time on self-study after a traditional lecture					
18	I find that time constraints and other commitments make it difficult to undertake self-study					
19	I struggle to access online learning materials					
20	I regularly use social media and online resources as part of my learning					
21	I find the use of technology in education daunting					
22	I have access to equipment and the internet to successfully watch videos and access learning materials					

What were the advantages of the flipped classroom model? What were the disadvantages of the flipped classroom model? What class activities would have been of value in a flipped classroom session? Do you prefer the flipped classroom or the traditional lecture? What are the reasons? Any other comments.

## Introduction

The flipped classroom model is a pedagogical model in which the typical lecture and homework/self-study elements of a course are reversed or ‗flipped‘ (Educause, 2012). Originating as part of the blended learning approach, the flipped classroom soon became a model in its own right. While still compared to blended, distance and online learning, there are important differences, as in the flipped model students still have face-to-face contact with tutors (Oblinger & Oblinger, 2005). Flipped learning requires careful preparation, with the need for flexible learning environments (Bergmann & Sams, 2012). Learning spaces may need to be rearranged to accommodate, for example, group work, performance or independent study. Staff need careful preparation and time to prepare learning materials (particularly in the early stages of implementation), and must be professional educators (Gojak, 2012). In addition, a shift in culture is required both from students and educators, as methods shift away from the lecture-centred instructional model (Hamdan, McKnight, McKnight, & Arfstrom, 2013). Lecturers, then, are no longer seen as bestowing information upon their students but are facilitators of education and take a more supportive role (Higher Education Academy, 2015).

In paramedic education it is essential that graduates possess the critical thinking and analytical skills that will enable them to become the autonomous and adaptable practitioners demanded by the role they will fulfil. The flipped classroom approach helps to develop such a skillset, as students are required to take more responsibility for their learning (Roehl, Reddy, & Shannon, 2013). The flipped approach when used in conjunction with virtual learning environments has been shown to be conducive to the acquisition of higher order critical thinking skills and increased creativity (Wallace, Walker, Braseby, & Sweet, 2014; Westermann, 2014). Undoubtedly, then, the flipped classroom approach has much value, particularly with regard to modules that teach practical skills on paramedic programmes (Alexander, 2015; Limmer, 2014). Such subjects benefit from a flipped approach as it allows more time in class for practice and demonstration of clinical paramedic skills. Students would be able to watch a video on, for example, intramuscular injection administration before class, have access to it while in class and devote their time in that session to learning and practising the practical skill instead of having to spend that time on learning underpinning theory. Studies show that the flipped classroom approach has met with a measure of success on paramedic education programmes, with clinical skills being thought to naturally lend themselves to this approach (Alexander, 2015).

Modules that teach student paramedics underpinning theoretical subjects, however, are a different matter. Anatomy, physiology, pathophysiology and pharmacology are very deep scientific subjects and it is as yet unclear whether it would be of benefit to teach these using a flipped approach. Although studies currently exist that examine the issues around using the flipped classroom model in paramedic education, these are either in practical areas (Alexander, 2015; Limmer, 2014) or do not specify what subject matter was actually taught using the flipped classroom (Phillips, 2016). Additionally, if the flipped classroom model is to be adopted across higher education (HE) paramedic programmes it is imperative that it is implemented because it has been found to be of real benefit to students, and not as a knee jerk reaction to increasing student numbers, class sizes and economic pressures (Young, 2015). With this in mind, an action research approach was taken to discover whether the adoption of a flipped classroom approach is suitable to teach student paramedics a theoretical subject. With use of the flipped classroom becoming more common in HE, it is vital to ensure it is employed where it can demonstrate real value in learning and teaching, rather than a wholesale approach being taken resulting in the model being adopted across the board.

## Methods

### Research design

This phenomenological study employed a mixed methods questionnaire design. The primary driver for choosing this method was comprehensiveness. The method allowed a wider range of issues to be addressed than a quantitative approach alone. While commonly used in health services research (Cohen, Manion, & Morrison, 2011), the value of this approach in educational research is being increasingly recognised in the literature (Ponce & Pagan-Maldonado, 2015). Discussion of this approach in a profession specific context is valuable, helping to strengthen the mixed methods research movement (Cresswell, 2009) and highlighting its strengths, challenges and possible uses (Ponce & Pagan-Maldonado, 2015).

### Sample

The study employed a convenience sample of a complete cohort of 20 full-time, second-year students already employed by a UK Ambulance Service as Emergency Care Assistants (ECAs). All participants were adult learners, with an equal number of male and female students and ages ranging from students in their twenties to those in their fifties.

### Ethical considerations

The university in which this study was conducted deemed that formal ethical approval was not required. Informed consent was obtained from all participants. An information sheet giving details of the proposed project was made available prior to commencement. A consent form was given to each prospective participant, which was signed and returned before the study commenced. Participants were aware that participation was completely voluntary and that even if they agreed to take part, they could withdraw at any time without prejudice. All data were anonymous, with questionnaires containing no identifiable information. No potential for harm was identified in the content or design of either the study or the questionnaire.

### Methods and data collection

A flipped approach was employed for a three-hour session on the physiology and management of pain that would normally have been taught by traditional lecture/seminar. Students were given a set of nine short videos, the longest of which was approximately 30 minutes and the shortest of which was approximately 9 minutes, to watch before attending class. The classroom time was devoted to activities designed to consolidate the information contained in the videos. The videos were not produced by the tutor but were of excellent quality, having been produced by a nurse lecturer at a university in the north of England.

After the session, participants completed a questionnaire of explanatory design, comprising both quantitative and qualitative elements (Supplementary 1). This design was chosen in order that the qualitative strand would give context and deeper meaning to results identified in the quantitative strand.

The quantitative section of the questionnaire was comprised of 22 closed questions, using a 5-point Likert scale. The questions addressed five major themes: educational style, flexibility, time, IT and responsibility.

The qualitative section comprised five open questions:

1. What were the advantages of the flipped classroom model?
2. What were the disadvantages of the flipped classroom model?
3. What class activities would have been of value in this case in a flipped classroom session?
4. Do you prefer the flipped classroom or traditional lecture? What are the reasons?
5. Any other comments.

### Data analysis

#### Quantitative

There is much debate as to whether Likert scale data should be treated as interval or ordinal data (Blaikie, 2003; Hansen, 2003; Pett, 1997). In this study, data were treated as ordinal as although response categories have a rank order, the intervals between them cannot be measured (Jamieson, 2004).

#### Qualitative

A general inductive approach was taken to the qualitative data analysis. An inductive approach is evident in much qualitative data analysis (Bryman & Burgess, 1994; Dey, 1993) and employs detailed reading of raw data to derive themes from the interpretations made by the researcher (Thomas, 2006). Qualitative data were analysed using thematic analysis, which is seen as a foundation method for qualitative analysis (Holloway & Todres, 2003). This method was chosen as it is relatively simple to use, offers flexibility and is ideal for novice researchers who may be unfamiliar with more complex methods of qualitative data analysis (Van Doorninck & Wong, 2012). It was vital in the process of qualitative data analysis that researcher presuppositions regarding the flipped classroom approach were acknowledged but then consciously suspended or ‗bracketed‘ in order to minimise reflexivity (Tufford & Newman, 2010). The entire data corpus was analysed for emerging patterns, and codes were manually produced. Data identified by the same code were grouped together and were then categorised into broad themes. The themes identified were:

1. Flexibility
2. Time
3. Access/preparation
4. Interaction
5. Learning styles/understanding

At this stage, respondent validation was undertaken by employing member checking. This was carried out by way of discussion with two participants to ensure they agreed with the interpretation of results and thematic analysis, thereby enhancing the trustworthiness of the study results (Birt, Scott, Cavers, Campbell, & Walter, 2016).

## Results

### Quantitative

#### Educational style

Of the students, 100% disagreed that the flipped classroom model was more engaging than a traditional lecture, while 80% agreed they would not recommend the flipped classroom to a friend.

When asked which method of delivery enhanced students‘ understanding the most, 85% said they found a traditional lecture made subjects easier to understand. Comparably, 70% disagreed that the flipped classroom better helped understanding of a subject.

Some contradictory data were produced regarding educational style. Of the students, 55% said they found class activities to be of more value to learning than a lecture, which contradicts the responses given to other questions. Results from this section are presented in Figure 1.

**Figure:**
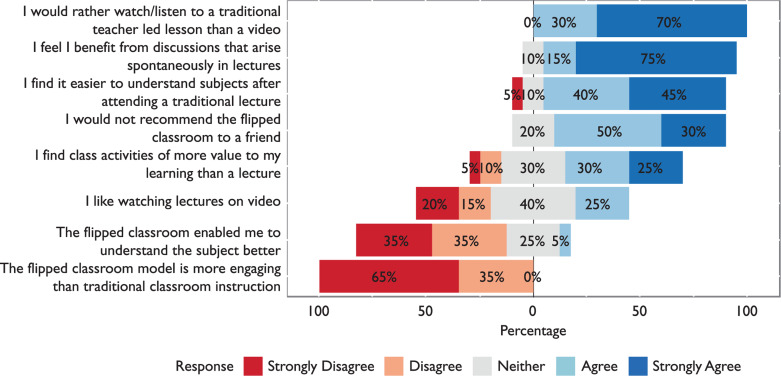
Figure 1. Students‘ educational style and preferred ways of learning.

#### Flexibility

Some contradictory data arose around the topic of flexibility of learning. Of the students, 60% agreed that they liked flexibility in their learning. However, when asked if a more structured approach was preferred, 70% agreed. A total of 20% neither agreed nor disagreed. Results in this section are presented in Figure 2.

**Figure:**
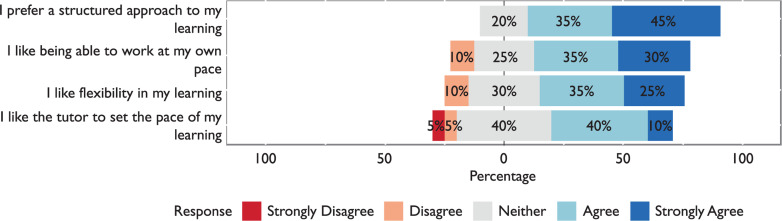
Figure 2. The importance of flexibility in learning to students.

#### Time

Time constraints were shown to be a definite concern, with 85% feeling that time constraints and other commitments made it hard for students to devote time to self-study. Students felt that the flipped classroom model compounded their time pressure, with 75% agreeing that it meant they were spending more time on self-study. Again, however, some contradictory data were produced here, as 45% of students agreed they were spending more time on self-study after a traditional lecture, with 15% agreeing and 30% strongly agreeing. A total of 40% neither agreed nor disagreed. Results in this section are presented in Figure 3.

**Figure:**
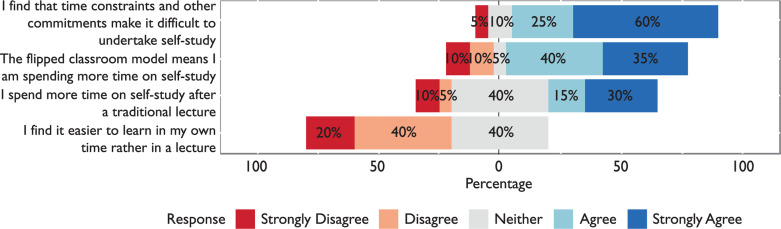
Figure 3. Time constraints and management.

#### IT issues

Of the students, 85% said they had access to IT equipment and the internet to successfully watch videos and access learning materials, while 50% disagreed that they struggled to access online learning materials and 40% gave a neutral response. While quantitative results show that on the whole there were no major IT issues, some data were produced regarding this area in the qualitative section of the questionnaire. Results in this section are presented in Figure 4.

**Figure:**
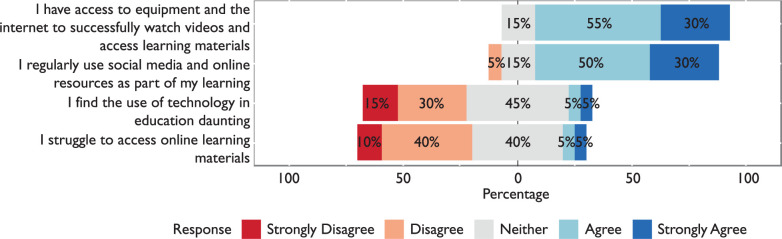
Figure 4. IT issues and confidence using technology in learning.

#### Responsibility

The questions designed to reflect how students felt about who was responsible for their learning gave broadly comparable results, with 35% of students agreeing that the lecturer was responsible, 40% giving a neutral response and 65% of students agreeing that they were in charge of their own education. Results in this section are presented in Figure 5.

**Figure:**
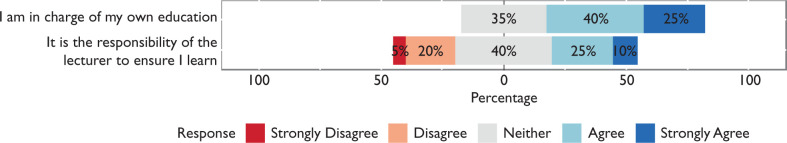
Figure 5. Responsibility for learning.

### Qualitative

#### Flexibility

As identified under the quantitative data section, students‘ perceptions of the term ‗flexibility‘ may have differed. One aspect of the flexibility of the flipped model that was extremely well received, however, was the ability to watch electures multiple times and to have the option to pause and rewind:

*I like the videos because I can watch in my own time and pause as needed. The ability to re-run a lesson is an advantage of the videos.*

Students also identified that the e-lectures made it easier than the traditional model to take notes:

*You are able to pause the flipped classroom to make own notes.*

Some students felt that it may be unnecessary in some cases to attend flipped classroom sessions:

*If you cannot attend a classroom lecture you are able to receive partial study at home.*

The feelings of the flipped classroom being a less personal approach could only be worsened by non-attendance.

#### Time

Students had real issues with time constraints and felt that the flipped classroom model meant their time was even more pressured due to an increased workload:

*Although you can watch at home it is still increasingly time consuming. Also it increases our workload as students especially if we have to watch videos then attend university to discuss.*

*When I am at university it is designated time that I can concentrate on what I need to learn. At home there are too many distractions and far too many other things I need to do to be able to focus on the e-learning videos.*

Additionally, students found that juggling self-study with work was also difficult:

*The main disadvantage was having time to watch all the videos. With all the other coursework plus working full time my time constraints were reduced.*

While it appears in this comment that the student has misunderstood the meaning of ‗constraints‘, the message is clear.

*Longer self-study, I work full time [for the ambulance service] and full time at uni. I struggle with finding time to access at work and home. It tips the balance of work/life.*

*From the point of view of someone who is not only a student but a full-time employee, time constraints are the biggest issue.*

#### Access/preparation

Having access to the e-lecture and other learning materials before attending a session was seen to be of value:

*[It] gives you an understanding of the subject before attending class.*

*It may give an individual a ‗head start‘ on a subject.*

The ability to access pre-session materials directly impacts upon the students‘ level of preparation. Some students did not access e-lectures due to time pressures:

*Although lectures are available on Blackboard I don‘t always read them.*

*I had very little time to watch the videos before the lecture.*

*I didn‘t have time to watch all the lectures.*

Other students identified other reasons regarding access that would affect levels of preparation:

*If the internet is poor it‘s very difficult to access.*

Failure to access pre-session materials for whatever reason will have a real effect on a student‘s ability to participate in sessions.

#### Interaction

Students were found to have missed the interaction of more traditional teaching methods:

*I need a teacher in front of me to ask questions. You cannot do this in front of a video.*

*I like dynamic lecturers with good knowledge who can answer questions – something that is missing in a flipped classroom.*

*It also lacks the facility to ask questions or clarify points not understood.*

As previously pointed out, the flipped classroom model still allows face-to-face contact with lecturers, so there the facility to ask questions exists; however, students made the following comments:

*Unable to ask questions as and when I need to and time to forget before classroom time.*

In contrast, regarding the traditional lecture/seminar method, students felt that:

*Everything is more personal. If you have a question you can get an instant reply. In the flipped classroom you can‘t.*

*Learning [is] more directed. Problems can be addressed at that time.*

Interaction with other students was also important and lacking in the flipped model:

*[I] miss out on other students‘ questions that also influence my own thought processes that I find boost my learning.*

*I learn from other people‘s questions in addition to my own. I do not hear the questions others ask in the flipped classroom.*

Opinions on what type of interaction is beneficial, however, can be mixed. With regard to spontaneous interaction in a traditional lecture/seminar, the following comment was made:

*I feel some of the off topic discussions that take place in a normal lecture are beneficial to my learning.*

Conversely, one student felt:

*The disadvantage of traditional teaching is distraction – ‗old ambulance tales‘ from tutors and peers.*

#### Learning styles/understanding

Disadvantages of the flipped model in this context were:

*People‘s differing perceptions of information learned.*

*You can watch a lecture at home, and in many cases, not understand what you are watching.*

It was interesting that students perceived the classroom session to be lacking in structure and organisation, particularly as a lesson plan was followed:

*[The] flipped classroom lacked structure and discipline.*

*Not well structured. No direction of learning or further learning/reading.*

*Also, I found the classroom work was interrupted as there was no structure and I feel the class was doing their own thing.*

A large number of students, while disliking the flipped classroom model, did enjoy the flexibility offered by e-lectures, and many suggested a blended approach would be extremely valuable:

*Lectures should be kept most definitely with occasional home videos.*

*I like a combination of both styles of learning.*

*A combination of flipped lecture and traditional lectures would be of benefit.*

*Useful as a secondary model post lecture.*

Students also liked the idea of tutor produced videos:

*Cannot a traditional lecture be podcasted?*

Ideas for improvement regarding activities were suggested:

*Maybe cross ref with added reading.*

*Answering MCQs following watching a few videos. Also labelling diagrams to assist with our learning.*

## Discussion

This study set out to explore whether students found the flipped classroom model a successful way to learn theoretical content on the subject of pain generation and management on a paramedic education programme. All students expressed a preference for the traditional lecture method rather than a flipped approach, with 85% agreeing that a traditional lecture made the subject easier to understand. It could be reasoned that the subject matter did not lend itself to a flipped approach; however, studies using the flipped approach in similar subject areas have found the model to have value (Tune, Sturek, & Basile, 2013). One explanation for this may be that learners entering HE from the Ambulance Service route tend to be older than those entering via the Universities and College Admissions Service (UCAS) entry and many have no previous experience of the self-directed, student-as-producer style of learning in HE. This is in some part due to their having been used to the old Ambulance Service didactic style of ‗training‘. The didactic ‗training‘ approach is teacher centred, where students take a passive role while tutors deliver information (New Learning, n.d.). This ‗training‘ differs greatly from ‗education‘ (Mortimer, 2015), and many ECAs find the transition from one to the other problematic, appearing to need ‗spoon feeding‘ rather than being able to take a more autonomous approach. Had this group had previous experience of HE they may have had a different perspective, as such approaches have been found to have more appeal to postgraduate students (Lameras, Levy, Paraskakis, & Webber, 2012). Individual differences in learning styles may also have been a reason for some contradictory data resulting in the educational styles section of the quantitative data.

Studies have already identified that feelings of disconnection have been reported by students regarding the flipped model (Campbell, Gibson, Hall, Richards, & Callery, 2008; Hanson, 2015; Johnston, Massa, & Burne, 2013), and this is comparable with the comments on interaction given by students in this study. Students felt that the ability to interact with a lecturer and peers was important, particularly with regard to the ability to ask questions, and this was seen to be a disadvantage of the flipped classroom. Previous studies have found that non-attendance was a potential consequence of the availability of online materials and that they may be seen as an alternative to attendance (Johnston et al., 2013). When this is in mind, feelings of disconnection and isolation can be compounded, particularly when trends towards decreasing student attendance have been identified due to online and electronic resources (Gupta & Susswein Saks, 2013).

Time constraints were considered a real issue and are a common criticism of the flipped model (Hanson, 2015; Post, Deal, & Hermanns, 2015). This had an even greater effect on this group of ECA students, who were already working full time. This result may have differed if the cohort had either been a mix of UCAS entry students and staff already employed by the Ambulance Service (as can be the case) or if it had consisted of UCAS students only. As the ECA students already work full time and are all adult learners with families, it is unsurprising that time was seen to be such an issue and this was highlighted by the results. The negative effects of paid employment on students‘ academic performance were highlighted by Lindsay and Paton-Saltzberg (1996), who found that reduced time available for study increased stress and decreased engagement. Additionally, Ford, Bosworth and Wilson (1995) found that these issues can be compounded further when that work involves a shift system. Not having time to watch all the videos or access them at all, as was the case with some students, impacts directly upon their ability to participate in the flipped session, and it would be interesting to see if non-employed undergraduate entry students would dedicate more time to pre-learning if working full time were not an issue. This appears to be a common problem with the flipped approach, with other studies finding that a lack of time resulted in students being ill prepared for the session (Hanson, 2015; Mikkelsen, 2015).

Despite students preferring the traditional seminar/lecture model overall, some aspects of the flipped classroom were very popular. The ability to pause and rewind the videos was seen as a definite advantage, and was perceived to make note taking easier. These results are directly comparable with the results of studies by Hanson (2015), Mikkelsen (2015) and Post et al. (2015). Flexibility was seen to be a positive aspect of the flipped classroom but again, some contradictory data arose here. This may be due to the students‘ perception of the word ‗flexibility‘, which could be viewed as subjective and mean different things to different people.

Students felt it would be advantageous to keep to the traditional model of lecture/seminar, but to make videos available online in addition to this as a support resource. One student suggested that podcasts of lectures could be provided, and it is worth considering whether tutor produced rather than generic online resources would have been better received. This would not be too onerous a task, with many universities having the facilities to capture lectures with inbuilt audio recording equipment. In the study by Post et al. (2015), criticisms were levelled at flipped approaches that used generic videos, with students seeing these as impersonal and bad value for money. This is a particularly important point in light of the loss of funding for paramedic students (Department of Health, 2017) and the move towards the consumer model of education by HE institutions (Eley & Stallman, 2014).

It was interesting that some students perceived the flipped classroom session to be unstructured, as a lot of thought and planning went into the format of the session. The structure of the flipped classroom session was challenging. While the session was not taking the didactic approach of a lecture, it was important that there was still structure, and it was quite difficult to find in-class activities that were relevant and informative. Of the studies that found the flipped classroom model to be a success, very few outlined how activities were chosen or what form these took. Additionally, it was important that the session included activities that reinforced learning objectives and encouraged students to use higher order thinking skills (Paulson & Faust, 1998), thus activities chosen were a mix of both individual and group tasks.

### Limitations

This study used a convenience sample, which means results are unlikely to be wholly representative of the population being studied. While a probability sampling method would have been preferable, this was not possible as access to participants was limited. Additionally, if convenience sampling were prohibited altogether due to questions of validity, some authors believe this would diminish the pool of legitimate data and slow scientific progress without justifiable cause (Landers & Behrend, 2015).

Volume of pre-learning materials was another limitation of this study. Due to the subject matter being so deep, the result was that students had to watch nine videos before attending the session. While the longest of these was only 30 minutes, it still resulted in approximately two hours of pre-learning being necessary. Studies have shown that pre-learning materials should be no more than 15 minutes long (Gilboy, Heinerichs, & Pazzaglia, 2015; Phillips, 2016). Taking the optimum length of pre-learning into account, however, it would have been very difficult to prepare students properly in this subject area so it may have been better if dedicated time for pre-learning had been allowed.

Choosing the right in-session activities and ensuring these were of the correct duration proved to be a challenge, particularly as little literature exists detailing how this can be achieved. Researcher inexperience of the flipped classroom approach had a bearing upon this, as content which would have usually taken up three hours of the session had already been delivered. The activities were a mixture of group and individual tasks, and on the group task where students were asked to discuss journal articles on the subject area, engagement levels decreased. This may have contributed to the students‘ perception of the session being disorganised. It may have been useful to test students‘ knowledge before and after both pre-learning and in-session activities.

## Conclusion

This study contributes to the literature surrounding the field of paramedic education and the move towards flipped classroom models and blended learning approaches by HE institutions. The flipped classroom undoubtedly has value when used in conjunction with virtual learning environments and a range of different tasks, and a blended approach was something students felt would be helpful. It is as yet unclear, however, whether the flipped classroom approach lends itself to complicated underpinning theoretical subjects, and further research is needed to explore a range of differing pre-session and in-session activities. Additionally, further research is needed to ascertain whether results would have been different had the sample been made up of student paramedics entering the programme via the UCAS route.

## Conflict of interest

None declared.

## Ethics

Not required.

## Funding

None.
